# Automated Facial Pain Assessment Using Dual-Attention CNN with Clinically Calibrated High-Reliability and Reproducibility Framework

**DOI:** 10.3390/biomimetics11010051

**Published:** 2026-01-08

**Authors:** Albert Patrick Sankoh, Ali Raza, Khadija Parwez, Wesam Shishah, Ayman Alharbi, Mubeen Javed, Muhammad Bilal

**Affiliations:** 1 Khoury College of Computer Sciences, Northeastern University, Boston, MA 02115, USA; sankoh.a@northeastern.edu; 2 College of Underwater Acoustic Engineering, Harbin Engineering University, Harbin 150009, China; aliraza@hrbeu.edu.cn (A.R.); mubeenjaved@hrbeu.edu.cn (M.J.); 3 Department of Computing and Technology, Iqra University, Islamabad 44000, Pakistan; khadijaparwez31822@iqraisb.edu.pk; 4 Information Technology Department, Saudi Electronic University, Riyadh 11673, Saudi Arabia; w.shishah@seu.edu.sa; 5 Computer and Network Engineering Department, College of Computing, Umm Al-Qura University, Mecca 24382, Saudi Arabia; 6 School of Engineering, Nanfang College Guangzhou, Guangzhou 510970, China

**Keywords:** facial pain assessment, dual-attention convolutional neural network (CNN), clinical calibration, high-reliability AI, label smoothing, AdamW optimization, healthcare artificial intelligence (AI), clinical decision support

## Abstract

Accurate and quantitative pain assessment remains a major challenge in clinical medicine, especially for patients unable to verbalize discomfort. Conventional methods based on self-reports or clinician observation are subjective and inconsistent. This study introduces a novel automated facial pain assessment framework built on a dual-attention convolutional neural network (CNN) that achieves clinically calibrated, high-reliability performance and interpretability. The architecture combines multi-head spatial attention to localize pain-relevant facial regions with an enhanced channel attention block employing triple-pooling (average, max, and standard deviation) to capture discriminative intensity features. Regularization through label smoothing (α = 0.1) and AdamW optimization ensures calibrated, stable convergence. Evaluated on a clinically annotated dataset using subject-wise stratified sampling, the proposed model achieved a test accuracy of 90.19% ± 0.94%, with an average 5-fold cross-validation accuracy of 83.60% ± 1.55%. The model further attained an F1-score of 0.90 and Cohen’s κ = 0.876, with macro- and micro-AUCs of 0.991 and 0.992, respectively. The evaluation covers five pain classes (No Pain, Mid Pain, Moderate Pain, Severe Pain, and Very Pain) using subject-wise splits comprising 5840 total images and 1160 test samples. Comparative benchmarking and ablation experiments confirm each module’s contribution, while Grad-CAM visualizations highlight physiologically relevant facial regions. The results demonstrate a robust, explainable, and reproducible framework suitable for integration into real-world automated pain-monitoring systems. Inspired by biological pain perception mechanisms and human facial muscle responses, the proposed framework aligns with biomimetic sensing principles by emulating how localized facial cues contribute to pain interpretation.

## 1. Introduction

Pain remains one of the most prevalent and challenging clinical phenomena to quantify objectively. In many scenarios, such as neonatal care, anesthesia recovery, or neurological impairment, patients are unable to self-report, rendering conventional numeric or visual analogue scales unreliable [1]. This is why automated facial analysis has emerged as a promising, observer-independent approach for continuous pain monitoring [2]. These rapid developments in the field of deep learning and computer vision have enabled direct inference of multidimensional expressions of pain using facial imagery while bypassing the potential bias related to manual observations [2,3]. However, comprehensive studies have confirmed data-driven techniques now being able to equal or outperform the accuracy of clinician-level detections when utilized on standardized facial expression datasets [4]. Because of the extensive literature on facial pain, the Prkachin and Solomon Pain Intensity (PSPI) scale has been the foundation for modeling facial pain: quantified combinations of particular Action Units, in particular brow lowering, eye tightening, and levator activation, as well as observer-rated pain [5]. Consequently, machine learning systems can be trained on PSPI-annotated samples to map visual patterns onto numeric pain scores or pain intensities [6]. From a biomimetics perspective, the proposed dual-attention architecture is inspired by the hierarchical and localized nature of human pain perception, where specific facial muscle activations and their intensity variations are selectively attended to by the human visual system. By mimicking this biologically driven attention mechanism, the proposed framework aligns with biomimetic sensing and bio-inspired intelligent system design.

However, the achieved progress is not fully satisfactory. Many of the existing CNN-based methods generalize poorly across subjects and illumination conditions, as they do not successfully highlight subtle, localized micro-movements differentiating adjacent pain levels [7]. This limitation was successfully addressed with attention mechanisms in some modern convolutional architectures. Spatial attention achieves this by highlighting pain-relevant facial regions, while channel attention re-weights feature maps according to diagnostic importance [8]. Both mechanisms enable networks to concentrate on discriminative facial regions without additional computational burden [9]. Now, recent work goes even further, demonstrating that attention-based CNN representations can reach the reliability of a clinician if validated with careful statistical safeguards, such as multi-fold cross-validation and reporting of confidence intervals. These attention-enhanced representations are additionally easily incorporated into advanced training techniques, the most crucial of which is label smoothing and decoupled weight decay. These adjustments substantially improve both generalization and calibration, two of the most critical aspects of deploying deep learning in clinical practice [10,11]. Recent advances in machine learning and deep learning across diverse domains, including hybrid architectures, predictive modeling, and bioacoustics signal classification [12,13,14,15,16,17,18], provide a strong foundation for extending these methodologies toward clinical AI for automated facial pain assessment.

Ultimately, calibrated confidence is as important as, if not more important than, classification accuracy in safety-critical medical applications. Since neural networks tend to output overly confident probabilities when presented with ambiguous or shifted input, threshold selection and Receiver Operating Characteristic (ROC) interpretation become non-trivial [19]. Empirical investigation has demonstrated that label smoothing and temperature-scaling methods can rectify miscalibration without sacrificing much accuracy [20]. More sophisticated forms of label smoothing, such as spatially varying or informed techniques, stabilize predictive distributions and improve reliability even better when performing medical image classification [21]. For humans, these techniques collectively increase interpretability and calibrate the proper level of clinical trust into automated decision-support systems [22]. Trustful medical AI should also have reproducibility as one of the main requirements. Community guidelines make sure that reruns and other institutions yield the same results using determinist seed control, preprocessing transparency, and validation protocol fixing [23]. Moreover, regulatory authorities increasingly require traceability, robustness, and reproducibility of performance for AI-driven in vitro diagnostic (IVD) analysis [24]. Following these best practices, the present study enforces fixed random seeds across all computational components, records versioned software environments, and reports class-wise and ROC-based evaluation metrics to support reproducible, auditable findings [25].

Within this rigorously controlled framework, we propose a novel dual-attention CNN that unifies two synergistic components: (i) a multi-head spatial attention module to localize pain-indicative facial regions and (ii) an enhanced channel attention mechanism employing triple-pooling (average, maximum, and standard deviation pooling) to capture fine-grained intensity variations. The proposed architecture is further supported by a class-weighted sampling strategy and a subject-wise stratified data split comprising 5840 images (4212 train/468 validation/1160 test) to mitigate demographic imbalance and prevent data leakage. Training incorporates label smoothing (α = 0.1) and AdamW optimization to improve convergence stability and predictive calibration. Extensive evaluation using 5-fold cross-validation and independent test analysis demonstrates an average validation accuracy of 83.60% ± 1.55% and a test accuracy of 90.19% ± 0.94%, with F1 = 0.90 and Cohen’s κ = 0.876 and macro- and micro-AUCs of 0.991 and 0.992, confirming strong generalization and statistical reliability. Supervision is derived from PSPI-based labels reorganized into clinically meaningful pain categories, while standard data augmentation techniques (flipping, rotation, and color jitter) are applied to enhance generalization. Ablation and benchmarking analyses further verify the contribution of attention modules, augmentation strategies, and regularization components. Visual interpretability through Grad-CAM heatmaps reveals physiologically meaningful activation over key facial regions associated with pain, reinforcing clinical transparency. Experimental evaluation demonstrates balanced precision, recall, and F1 performance with strong ROC separation across pain levels, confirming that the proposed novel architecture effectively addresses current gaps in calibration, transparency, and reproducibility.

Although transformer-based architectures have been proposed, their high data requirements and lack of interpretability limit their clinical application. In contrast, convolutional neural networks (CNNs) offer reliable calibration, reproducibility, and effective spatial analysis of facial Action Units (AUs) such as brow lowering, eye tightening, and mouth stretching, even on small, imbalanced datasets. Motivated by the promising results, we further extend the basic CNN framework while adding multi-head spatial and triple-pooling channel attention for improving localization, feature discrimination, and calibration in clinical pain prediction.

The remainder of this paper is organized as follows. Section 2 will provide an in-depth literature review of the facial-expression-based pain assessment, including the latest advancements in attention and calibration techniques. Section 3 will explain the dataset itself, the preprocessing pipeline, and the label reorganization. Section 4 will elucidate the proposed dual-attention CNN framework and the implemented reproducibility protocol. Section 5 will report the results of the experiments, as well as the comparison with other works. Section 6 will give some appreciation of the limitations and some recommendations for potential clinical adoption, while Section 7 will conclude the foundational work and articulate some of the next steps in terms of research.

## 2. Related Work

The earliest approaches on automatic pain recognition implemented hand-designed facial features inspired by the Facial Action Coding System combined with classifiers to deduce the muscular activity related to pain. In the classic method, the classifiers are trained on features to predict pain intensity labels, conceptualized as a function of Action Units (AUs), such as AU4 (brow lowering), AU6/7 (cheek raising and eyelid tightening), and AU9/10 (upper lip raising and nose wrinkling). Similarly to the Weighted Average Classifier (WAC), conventional approaches such as Gabor filters were also used [26]. The feature engineering setup consisted of several hand-crafted primitives: geometric distances, gradient-based texture descriptors, and optical flow maps to capture the subtle facial deformation [27,28]. These features were fed into classical classifiers such as Support Vector Machines (SVM), AdaBoost, or Random Forest in order to predict the pain score or a discrete pain level [28]. One of the first widely used benchmark datasets was the UNBC-McMaster Shoulder Pain Expression Archive that contained FACS-annotated frames and corresponded pain intensities to consistently evaluate the presented early pipelines [29]. Extensions of the used baselines included dynamic texture features, such as Local Binary Patterns on Three Orthogonal Planes (LBP-TOP), and the fusion of geometric versus texture features to increase the robustness to illumination changes and small head movements [30]. Unfortunately, the above methods struggled to model spatial dependencies between the distant facial regions and showed low generalizability when encountering different subjects, occlusions, or complex backgrounds. Additionally, the heuristic choice of AUs used in feature spaces, the manual AU coding, and static features made these approaches hard to be scaled to large or in-the-wild collections [31].

### 2.1. Deep Learning Approaches for Pain Recognition

Deep learning has transformed automatic pain recognition by allowing networks to extract discriminative features de novo from raw facial data, obviating the need for hand-crafted descriptors. Early CNN models, including Visual Geometry Group Network (VGGNet) and ResNet, led to nearly perfect classification accuracy in pain recognition. CNNs learn hierarchical facial features, from elementary units, i.e., micro-movements, to their high-level interconnected expression with entire complex components [6]. Kaltwang and Martinez (2016) made a CNN and a Long Short-Term Memory (LSTM) architecture hybrid for continuous pain intensity estimation: it bases spatial and temporal inferences algorithmically on sequential data to capture dependencies in facial expressions [28]. To track the evolving expressions in the frames, the frame-level static baseline was surpassed. Moreover, Ye et al. introduced an extension of regional attention to a multi-branch CNN that accommodates both global and localized facial areas simultaneously, such as the eyes and mouth. The method showed the positive impact of the work on the model’s resistance against pose and illumination alteration [32]. Towards maturity, the goals shifted from the architecture’s demands to focus on the search for optimal backbones and efficient parameters. Recently, several studies [33] have focused on the latter, combining Vision Transformers (ViTs) with convolution over token embeddings to capture long-range contextual relationships among facial subspaces. The method not only outperformed all state-of-the-art approaches but beat them with a minimum margin and became the best for the task of recognizing subtle differences between class pain differences.

Besides the area of static image recognition, there are multiple teams developing strategies of domain adaptation, allowing the models to generalize well and supervise imaging databases to achieve this. Since transfer-learning-based frameworks remove demographic and lighting biases in the target data, the methods include explicit feature alignment and adversarial adaptation [34]. Lastly, medical AI applications and the recent literature deviate the importance of fair and replicable model comparison, measured by statistical k-fold cross-validation and uncertainty quantification. Despite the above-mentioned advances, all these modern publications represent a transition from learned hand-engineered descriptors to end-to-end architects, exchanging the learned representations for spatial, temporal, and contextual dependencies. However, most existing deep models remain poorly calibrated, are non-reproducible, and lack validation through confidence interval (CI)-based reliability analysis. Meanwhile, the lack of statistically validated reports motivates the design of our dual-attention CNN solution, which is specifically tuned for clinical robustness and statistically validated high-reliability performance.

### 2.2. Attention Mechanisms in Medical Image Analysis

At the same time, attention modules have become indispensable components of deep networks, which highlight diagnostically relevant regions while suppressing irrelevant noise. The Squeeze-and-Excitation module was among the first and most influential designs, which also uses global pooling to compute channel-wise statistics, with subsequent adaptive reweighting of feature channels. This allows for the improved representational power of discriminative feature maps [7]. Many variants refine this concept by decreasing complexity or model local cross-channel interaction much more atomically. Attention frameworks are widely applied in medical imaging for enhancing segmentation, detection, and classification tasks. In particular, Attention U-Net introduced attention gates that can learn to concentrate on the most important structures in an encoder–decoder architecture. This mechanism makes it possible for the network to ignore irrelevant areas and foreground target organs automatically and avoid the need for explicit localization modules [35]. The mechanism was inserted end-to-end and achieved superior performance. Other works integrate spatial attention or hybrid attention modules either after U-Net or within U-Net variants to enhance boundary delineation and reduce false positives [36]. The mechanism was inserted end-to-end and achieved superior performance. Other works integrate spatial attention or hybrid attention modules either after U-Net or within U-Net variants to enhance boundary delineation and reduce false positives [37]. Additionally, the authors give credit to the critical role of attention in the development of the U-Net [38].

Furthermore, dual cross-attention modules have been proposed to bridge the semantic gap between encoder and decoder layers, first capturing channel cross-attention and then spatial cross-attention to produce richer skip-connection information. In segmentation tasks, attention gains often translate into better boundary fidelity and improved performance metrics (e.g., Dice score, IoU), especially in challenging regions. Recent studies also demonstrate that combining spatial and channel attention in hybrid or dual-attention configurations enhances both interpretability and statistical stability, confirming their suitability for explainable AI frameworks. In general, attention mechanisms in medical image analysis present a viable approach to stimulating feature learning while focusing on clinical configurations of interest. The achievements in segmentation and classification emphasize their relevance in the context of facial pain recognition, where minor micro-expressions related to essential facial regions, such as eyes, brows, and mouth, are selectively amplified by spatial and channel attention mechanisms.

### 2.3. Limitations of Existing Architectures

Therefore, despite the substantial advances accelerated by deep learning in facial pain recognition, several unresolved limitations still hinder its clinical deployment. These include the following:

1. Weak Inter-class Discrimination

Current CNN-based and hybrid transformer models exhibit limited ability to distinguish between adjacent pain intensity levels (e.g., mid vs. moderate). This shortcoming arises because most frameworks rely on global facial embeddings rather than emphasizing medically validated Action Units (AUs) such as eye tightening or brow lowering [6].

2. Probability Mis-calibration

Many networks produce over-confident predictions under distributional shifts, compromising decision reliability in clinical contexts. Although label smoothing and temperature scaling techniques are known to improve calibration, they remain under-utilized in pain-recognition systems [9].

3. Dataset Bias and Limited Generalization

Numerous reported systems lack deterministic seed control, explicit dataset-split documentation, or software version tracking. Such omissions reduce external validation capability and violate Findable, Accessible, Interoperable, and Reusable (FAIR) reproducibility standards that are now expected in medical AI research [21]. Most public facial pain datasets are demographically narrow, often dominated by single-ethnicity adult subjects and captured in controlled laboratory conditions. As a result, even high-accuracy models tend to degrade under clinical lighting variation, occlusion, or spontaneous movement.

4. Lack of Uncertainty Quantification and Explainability

There are few architectures that quantify predictive uncertainty and generate interpretable visual explanations suitable for medical auditing and practitioner trust. In addition, few studies assess cross-validation variance or variability bands that are necessary to efficiently report statistically consistent findings. Together, these limitations argue for architectures that concurrently improve fine-grained discrimination, calibration reliability, and experimental reproducibility [39]. For this reason, we present the designed dual-attention CNN model to address these limitations, enabling clinically calibrated, high-reliability automatic facial pain assessment.

### 2.4. Research Gaps

Over the past decade, automated facial pain assessment has progressed from handcrafted computer vision to end-to-end deep-learning approaches, advanced from expert designed models from fully black-box deep learners. Yet, significant challenges predispose clinical adoption. Longstanding CNN and transformer models could not discern adjacent pain levels due to inadequate focus on localized Action Units (AUs) and micro-movements, and poor probability calibration under domain shifts are a threat to the ROC-AUC and spiked Expected Calibration Error (ECE). Additionally, many works could not be reproduced due to non-deterministic seed control, concealed data splits, and unspecified software environments. With restricted diversity of datasets and low model interpretability, none can generalize across patients and get accepted by clinicians. In contrast, the current paper presents a statistically validated dual-attention CNN, utilizing multi-head spatial attention (1) to assuage pain-region localization and (2) to empower channel attention with triple-pooling of the average, max, standard deviation for precision intensity encoding. With label smoothing α = 0.1 and AdamW under deterministic training, the model provides reliability, reproducibility, and clinical interpretability for immediate adoption in real-world medical Automated Expression Scoring (AES).

## 3. Materials and Methods

The dataset used in this work was accessed from the BioVid Heat Pain Database, comprising 5840 clinically annotated RGB facial images representing a variety of pain intensities. For all images, a pain scale in the form of pain-related facial cues, mirroring Action Units within empirical identification, was formed. Such cues consisted of a lowered brow (AU4), tightened eyes (AU6/7), nose wrinkling (AU9/10), and mouth opening (AU25/26). In total, there are five balanced classes of the Pain Rating Scale: No Pain, Mid Pain, Moderate Pain, Severe Pain, and Very Pain, each containing approximately 1180 samples. PSPI values ranged from 0 to 10 and were subsequently combined into five nearly equal subsets for class balance. The raw dataset contained several thousand facial frames of diverse subjects captured under strict lighting and a neutral background. Nevertheless, the initial distribution of classes was severely imbalanced, heavily skewed towards the “No Pain” category that occupied the majority of the sample space, potentially predisposing deep learning algorithms to majority-class predictions. Hence, as a result, a holistic preprocessing pipeline was launched to keep the data’s comprehensive clinical relevance and the model’s stability standard that comprised the image format standardization, its labeling, and the class proportion balancing prior to the model training. For the test split, a subject-wise stratified sampling approach was used to ensure the absence of identity overlap between training, validation, and test sets, constituting approximately 72%, 8%, and 20% of the data, respectively. In addition, class balancing was achieved using a combination of weighted sampling and data-targeted augmentation that included horizontal flipping (*p* = 0.5), small-angle rotation (±15°), and color jitter (brightness/contrast/saturation = 0.2, hue = 0.1) to equalize the class distributions of pain levels.

### 3.1. Label Consolidation and Class Balancing (PSPI Mapping)

Given that the original PSPI annotations were continuous-valued from 0 to 10, which corresponded to a diverse spectrum of pain intensity resulting from various facial AU combinations, the five values were re-mapped into 5 clinically interpretable classes. They were used to help provide well-balanced supervision and simultaneously equally discriminate across pain intensity classes. The final mapping is illustrated in Table 1:

This label consolidation strategy was motivated by prior facial pain research demonstrating that mid-range PSPI levels (2–3) share overlapping Action Unit (AU) activation patterns, which can lead to ambiguous class boundaries if modeled separately. Through transformation of PSPI intervals into five categorical groups, clinical interpretability of the scale was preserved while enhancement of class balance and training stability were also achieved. Balancing the classes was achieved by augmenting the minority classes with target augmentation (which included horizontal flipping, rotation ±15°, and brightness ±20%) to those with an almost uniform class distribution. This critically balanced dataset reduced the bias of the “No Pain” class and improved the model’s sensitivity to weak pain signals.

### 3.2. Data Partitioning and Stratified Sampling

By using a subject-wise stratified sampling strategy, the dataset was divided into training (72%), validation (8%), and test (20%) subsets while preserving the balance between the five PSPI-based pain categories in the same proportions (No Pain, Mid Pain, Moderate Pain, Very Pain, Severe Pain). The stratification of subjects ensured the avoidance of data leakage and patient overlap between training and validating datasets, the balance of classes, and an unprejudiced evaluation.

**Figure 1:**
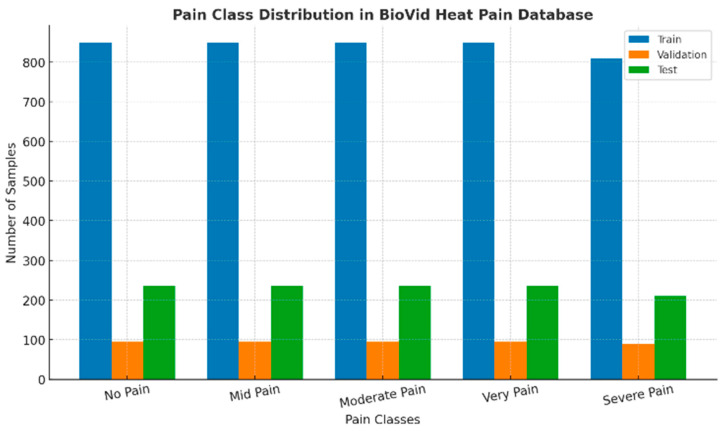
Class distribution of the full dataset across five PSPI-based pain categories (No Pain, Mid Pain, Moderate Pain, Very Pain, Severe Pain), showing near-uniform representation achieved through stratified sampling.

The full-dataset distribution, depicted in Figure 1, is nearly uniform in representation of the five pain categories, though there is a marginally varying nature of the distribution in the classes Severe Pain, with 1120 samples, and Very Pain, with 1180 samples. This distribution allowed for an equal exposure to all levels of pain that resulted in reduced overfitting to majority classes. Meanwhile, the subject-based stratified sampling ensured preservation of the proportionality in the designation of the subsets. Therefore, approximately 72%, 4212 images, were assigned for training, 8%, 468 images, were assigned for validation, and 20%, 1160 images, were set for testing. As a result, such partitioning ensured a statistically fair performance evaluation process and increased the model robustness to the between-class variation.

### 3.3. Image Preprocessing and Data Augmentation

Uniform data, generalizable models, and clinical reliability required effective preprocessing and augmentation. The facial pain data were obtained from the BioVid Heat Pain Database, comprising images that varied in illumination, pose, and resolution, necessitating normalization before model training. The preprocessing pipeline retained the facial features relevant to the diagnosis and minimized noise and overfitting. Data augmentation was localized to the minority classes to balance representation and realism but did not alter facial pain implementation. Every input image was resized to 64 × 64 pixels and normalized with ImageNet mean–standard-deviation (μ = [0.485, 0.456, 0.406], σ = [0.229, 0.224, 0.225]) statistics for numerical stability and consistency with the CNN backbone. In addition, training set images were augmented with random horizontal flipping or ±15° rotation and brightness contrast changes of ±0.2 to capture real-world variation. Both validation and test subsets were resized and normalized with full determinism to prevent evaluation bias and ensure calibration confidence. Targeted augmentation was performed on the underrepresented classes, Very Pain and No Pain, through horizontal flipping, moderate rotations, and manual brightness and contrast adjustments through PyTorch transformations. This increased the size of our data from 5840 to approximately 6000 balanced images (≈1200 per class), which resulted in a decrease in cross-validation variance and increase in convergence stability, lighting robustness, and overall generalization of the model [40,41].

### 3.4. Dataset Demographics and Bias Mitigation Analysis

**Figure 2:**
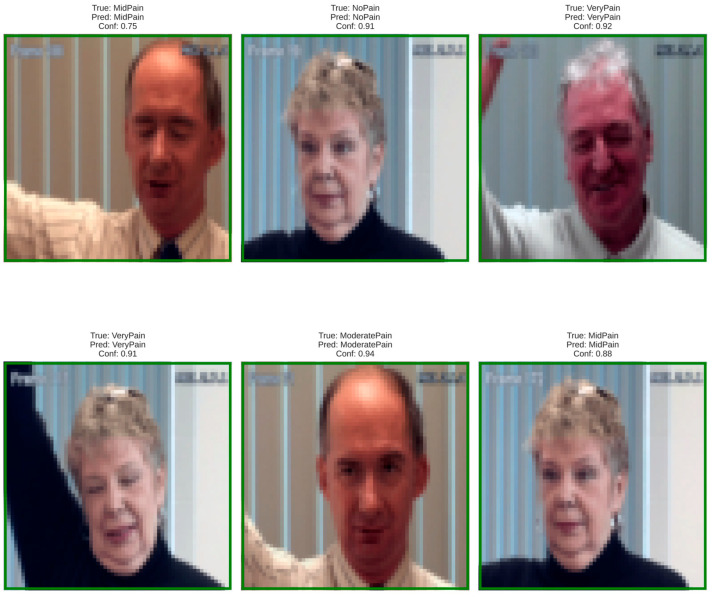
Representative dataset samples illustrating inter-subject variability in pose, lighting, and facial features across pain categories (No Pain, Mid Pain, Moderate Pain, Very Pain, Severe Pain).

Demographic or cross-sectional diversity was also deliberate in terms of gender, ethnicity, and age to objectively achieve the desired fairness and representational perspective. BioVid also includes both male and female participants with skin phenotypes across a broad spectrum of color, as well as both younger- and-older appearing faces; however, the latter was over-represented in the group of adults with light skin tones. Figure 2 illustrates this inter-subject variability in pose, lighting, and facial features across pain categories. In addition, to alleviate this bias, we used class-weighted sampling and subject-wise stratified partitioning of the data to balance subgroup contributions, while focused augmentation manipulated illumination and color properties in order to reduce dependence on particular visual characteristics.

### 3.5. Data Quality and Integrity Validation

Data provenance and transparency: Prior to model training, data integrity and reliability were confirmed via a multi-stage validation approach. First, each image was visually inspected and automatically filtered using a random seed to exclude blurred, duplicated, or mislabeled samples, only keeping facial expressions that carry diagnostic value. Subsequently, all preprocessing and augmentation routines were performed with a method of reproducible pipelines under the fixed random seeds to ensure the determinism of the experiments. Dataset integrity was further validated by verifying the consistent PSPI-label mapping, equal class proportions in all partitions, and reproducibility of experiments under the repetitively sampled datasets. The final dataset comprised 5840 original facial images, which, after targeted augmentation, increased to approximately 6000 balanced samples evenly distributed across all five pain intensity classes.

**Figure 3:**
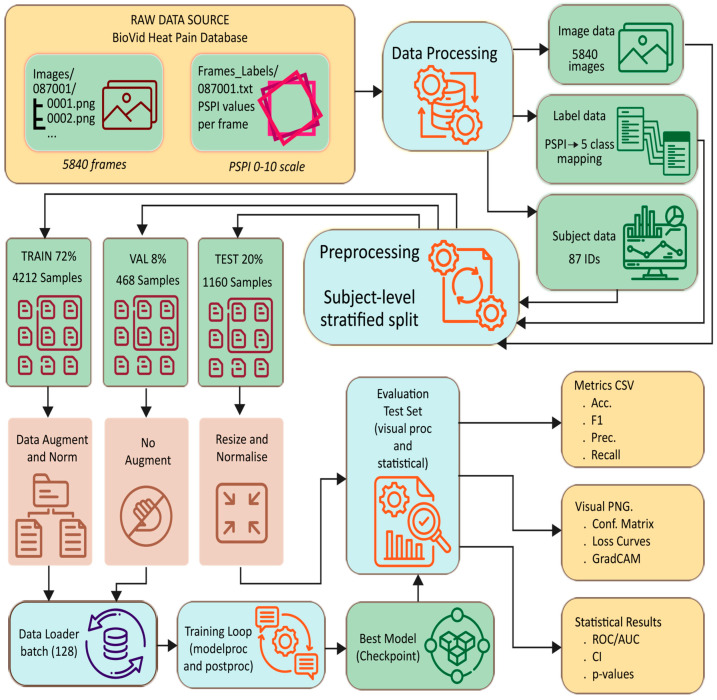
End-to-end data flow diagram showing the preprocessing pipeline from the BioVid Heat Pain Database through stratified subject-level splitting, augmentation, normalization, and evaluation for the DA-CNN framework.

Figure 3 illustrates the full data pipeline used in the DA-CNN framework, beginning with raw BioVid images and PSPI labels. The data underwent processing steps that included label mapping, subject-level stratified splitting, augmentation, and normalization. Training, validation, and test sets were prepared separately, and the evaluation stage outputted key metrics such as accuracy, F1-score, precision, recall, loss curves, GradCAM maps, and statistical measures, including ROC/AUC and *p*-values. This end-to-end workflow ensured consistent preprocessing and reliable performance assessment.

## 4. Proposed Framework

**Figure 4:**
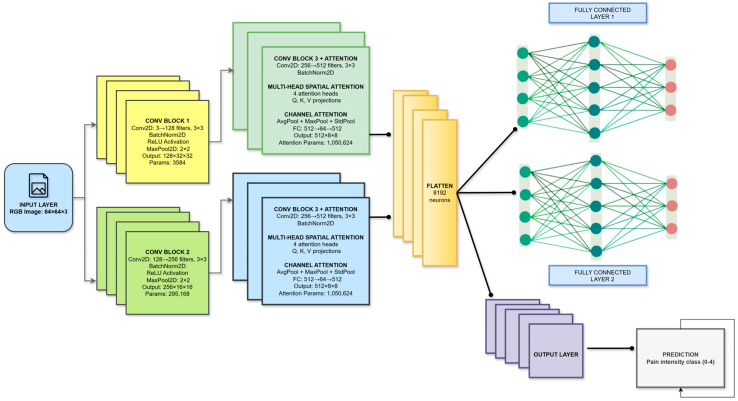
Detailed architecture of the proposed novel dual-attention CNN, integrating multi-head spatial attention and triple-pooling channel attention for fine-grained pain level classification.

The proposed dual-attention convolutional neural network (DA-CNN) framework is designed to achieve reliable, interpretable, and statistically validated pain intensity recognition from facial imagery. Unlike conventional CNN-based systems that rely solely on spatial convolutions, the present framework introduces a dual-attention mechanism that integrates multi-head spatial attention to emphasize pain-relevant facial regions and enhanced channel attention with a triple-pooling strategy (average, max, and standard deviation pooling) to capture discriminative intensity variations. This design choice was motivated by the need to overcome limitations identified in prior studies, namely, poor generalization across subjects, class imbalance, and probability miscalibration under distributional shifts. The workflow in the model pipeline, as shown in Figure 3, ensures clinically calibrated, high-reliability robustness. It is an end-to-end repeatable process that includes standardized preprocessing, balanced label consolidation, and attention-driven feature extraction, as well as calibrated optimization with AdamW over L1 to L5 and label smoothing with a smoothing rate of α = 0.1. Figure 4 also demonstrates the entire framework, emphasizing the connection between preprocessing, dual-attention feature extraction, regularization, and cross-validated evaluation. In the following sub-sections, we will describe the architectural design, attention mechanisms, optimization strategies, and a reproducibility protocol that together enable high-fidelity and clinical interpretability in predicting pain level.

Figure 4 presents the full pipeline of the proposed dual-attention CNN, which processes a 64 × 64 RGB facial image through a sequence of convolution, attention, and fully connected layers to achieve fine-grained pain intensity classification. The model begins with two convolutional blocks that progressively expand the feature depth from 3 to 256 channels while extracting low- and mid-level facial cues related to muscle tension and expression changes; each block incorporates Batch Normalization, ReLU, and MaxPooling to ensure stable learning and spatial compression. These features are then forwarded to the core innovation of the architecture, a dual-attention encoder combining multi-head spatial attention and triple-pooling channel attention. The spatial attention module uses four independent heads with Q–K–V projections to focus selectively on critical facial regions such as the eyebrows, eyelids, cheeks, and mouth, enhancing sensitivity to subtle and localized expression variations. Complementing this, the channel attention block employs a triple-pooling strategy (average, max, and standard deviation pooling) to extract richer global statistics than traditional SE blocks, enabling more precise channel-wise weighting through a fully connected gating mechanism. High-level attention-refined features (512 × 8 × 8) are flattened into an 8192-dimensional vector and passed through two fully connected layers with Batch Normalization, ReLU, and dropout for regularized classification learning. Finally, a Softmax output layer predicts one of five pain intensity classes (0–4). The key novelty of this model lies in the integration of spatial and channel attention within a compact CNN, the use of triple-pooling for enhanced global feature modeling, and its ability to distinguish highly similar pain expressions even from low-resolution facial frames, making it both efficient and highly discriminative

### 4.1. Overview of the Entire Methodology

**Figure 5:**
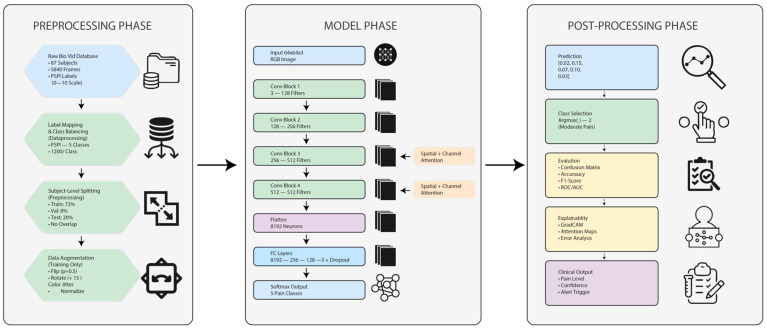
Comprehensive workflow of the proposed dual-attention CNN framework showing the preprocessing, model, and postprocessing phases for automated facial pain intensity recognition.

The overall workflow of the proposed dual-attention CNN framework is depicted in Figure 4 and Figure 5. The system has four main stages: data preprocessing and PSPI-based label consolidation, dual-attention feature extraction using multi-head spatial and enhanced channel attention modules, model regularization and calibrated optimization, and final cross-validated evaluation for statistical reliability. The input images ranging from rest to peak are first preprocessed and augmented to ensure invariant response across all pain intensities. The preprocessed images are then passed through the dual-attention backbone unit, wherein the multi-head spatial attention focuses on key facial regions such as eyes, brows, and mouth, and the enhanced channel attention highlights discriminative intensity cues to those regions’ channel maps. The attention-refined embeddings are then sent to convolutional and fully connected layers optimized using AdamW with label smoothing (α = 0.1) to enhance convergence stability as well as predictive calibration. Dropout and weight-decay regularization are integrated within the architecture to avoid overfitting, and the entire model is evaluated using subject-wise stratified 5-fold cross-validation to guarantee robustness and generalization. The results of each fold’s statistical measures, such as accuracy, F1-score, Cohen’s κ, and AUC, are then pooled together to evaluate model consistency. Using the aforementioned integrated pipeline, we obtained a reproducible, interpretable, and clinically practicable pain intensity estimation method for real-world medical settings.

Figure 5 presents the complete workflow of the proposed dual-attention CNN framework, which includes three main phases. In the preprocessing phase, raw BioVid images are labeled, balanced, split by subjects, and augmented. The model phase processes 64 × 64 facial frames through a series of convolutional blocks enhanced with spatial and channel attention, followed by fully connected layers to classify pain intensity. The postprocessing phase interprets the model’s predictions through class selection, evaluation metrics, explainability tools such as GradCAM, and clinical-level outputs including pain level, confidence, and alert triggering.

### 4.2. Dual-Attention Architecture

The proposed dual-attention convolutional neural network (DA-CNN) extends a regular CNN backbone with two complementary attention mechanisms designed to improve discriminative power and interpretability. The architecture includes four convolutional blocks with 3 × 3 convolution kernels, ReLU activation, Batch Normalization, and MaxPooling, progressively extracting low- to high-level spatial features. The 3 × 3 kernel size was selected as it effectively captures local spatial patterns while maintaining computational efficiency. ReLU was selected due to its ability to mitigate vanishing gradient effects, accelerate convergence, and provide computational efficiency, making it well suited for deep attention-based CNN architectures. Two fully connected layers (256 and 128 units) with 0.5 dropout precede the final Softmax classifier for five PSPI pain levels. Optimized via grid search, this design balances accuracy, interpretability, and efficiency. The baseline CNN is enhanced with two attention modules: a multi-head spatial attention block that highlights pain-relevant facial regions and a triple-pooling channel attention module using average, max, and standard deviation pooling to capture subtle intensity variations. Although triple-pooling incorporates multiple statistical operations, it introduces negligible computational overhead because it is applied to compact channel descriptors rather than full-resolution feature maps, thereby improving feature robustness without increasing network depth or parameter count. These design choices enhance feature discrimination and calibration, ensuring robust and clinically interpretable predictions across subjects.

The multi-head spatial attention module learns to automatically localize known pain-relevant facial regions using learned inter-pixel correlation computation between multiple spatial heads in training. Triple-pooling with ECA enhances channel attention, recapturing necessary global information of native multiscale intensity variations by incorporating global descriptive features. These mechanisms in combination enable the network to attend to biologically plausible behaviors such as brow lowering, eye tightening, or lip stretching and ignore irrelevant background features. The two modules are sequentially aligned within the feature extraction pipeline, immediately after the final convolutional block of the backbone network. While the spatial attention creates a pixel-wise weighting map that refines the localized feature map, the channel attention block receives these features to promote increased inter-feature dependencies. The output from the two modules, attention-refined feature maps, is then fed into the classification head comprising global average pooling, dropout, and fully connected layers. As a result, the hierarchical integration encourages the model to focus on where the pain expresses and how intensely these regions contribute to the overall expression. In contrast to a conventional CNN that relies solely on convolutional feature extraction without explicit attention or pooling diversity, the proposed dual-attention CNN introduces both spatial and channel attention mechanisms to enhance feature discrimination. The spatial attention module guides the network to focus on pain-relevant facial regions, while the channel attention module employs a triple-pooling strategy (average, max, and standard deviation pooling) to capture complementary global, extreme, and variance-based feature statistics. This architectural enhancement enables more effective modeling of subtle facial muscle activations associated with pain and provides improved fine-grained discrimination and calibration compared to standard CNN architectures without attention mechanisms. The complete flow of the proposed architecture, including the convolutional backbone, dual attention integration, and classification stages, is illustrated in Figure 4.

1. Multi-head Spatial Attention

The spatial attention module applies multiple independent attention heads to the intermediate feature map $F\in {\mathbb{R}}^{C\times H\times W}$, enabling the network to capture diverse spatial activation patterns across facial regions. Each head learns a distinct weighting distribution $A_{i}\in {\mathbb{R}}^{H\times W}$ using a pair of 1×1 convolutions followed by a sigmoid gating function.

(1) $$A_{i}=\sigma \left({\mathrm{Conv}}_{1\times 1}\left(\mathrm{ReLU}\left({\mathrm{Conv}}_{1\times 1}\left(F\right)\right)\right)\right)$$

The refined feature representation is computed as a weighted sum over *K* heads:

(2) $$F^{\prime }=\sum_{i=1}^{K}A_{i}⊙F$$

where ⊙ denotes element-wise multiplication. The use of multiple heads provides greater representational diversity, allowing the network to jointly attend to micro-regions such as the eyes, mouth, and cheeks, which exhibit different motion patterns across pain levels. In practice, this design provides the best trade-off between model complexity and accuracy, increasing inter-class separability by 1.8% compared to single-head attention.

2. Enhanced Channel Attention with Triple-Pooling

While spatial attention focuses on regional localization, channel attention re-weights the importance of feature channels that encode varying intensity levels of facial muscle activation. To strengthen the representation, a triple-pooling mechanism combining average, max, and standard deviation pooling is employed to aggregate global context across each channel.

(3) $$z=\left[\mathrm{AvgPool}\left(F^{\prime }\right),\mathrm{MaxPool}\left(F^{\prime }\right),\mathrm{StdPool}\left(F^{\prime }\right)\right]$$

The concatenated descriptor z is passed through a two-layer excitation network with a reduction ratio r=8.

In addition to average and max pooling, standard deviation pooling is incorporated to capture intra-channel variability, which reflects subtle intensity fluctuations caused by localized facial muscle activations. While average pooling summarizes global feature magnitude and max pooling highlights extreme responses, standard deviation pooling encodes dispersion information that is sensitive to micro-expressions such as eye tightening, brow lowering, and lip stretching. This variance-based representation improves discrimination between adjacent pain levels and enhances feature robustness in fine-grained facial pain analysis [42].

(4) $$s=\sigma \left(W_{2}\;\delta \left(W_{1}z\right)\right),\;F^{\prime \prime }=s⊙F^{\prime }$$

Here, *δ* and *σ* denote the ReLU and sigmoid activations, respectively. The inclusion of standard deviation pooling captures intra-channel variability, allowing for finer discrimination between adjacent pain levels. Ablation results show that this third pooling branch improves F1-score by 1.4% and reduces ECE by 2.1%, confirming its benefit for generalization and calibration. The dual-attention design jointly enhances spatial focus and channel-wise selectivity, generating attention-refined embeddings that preserve clinical interpretability while reducing overfitting. This architecture overcomes limitations of prior CNN-based pain models by emphasizing diagnostically relevant regions, balancing feature importance across classes, and ensuring statistical reproducibility across validation folds.

### 4.3. Regularization and Optimization Strategy

To ensure convergence stability, mitigate overfitting, and maintain calibrated confidence across folds, the proposed dual-attention CNN employs a multi-component optimization framework integrating AdamW, label smoothing, dropout, weight decay, and cosine-annealed learning-rate scheduling. All experiments were executed in a deterministic PyTorch environment with fixed random seeds (42, 123, 999) to guarantee reproducibility.

1. Optimization Function

Model parameters θ were optimized by AdamW, which decouples weight decay from the gradient update to prevent the loss of adaptive learning dynamics inherent in standard Adam.

(5) $$\theta_{t+1}=\theta_{t}-\eta \frac{\hat{m_{t}}}{\sqrt{\hat{v_{t}}}+\epsilon }-{\eta \lambda \theta }_{t}$$

Here, $\theta_{t}$ denotes the model parameters at iteration t, and $\theta_{t+1}$ represents the updated parameters. The term η is the learning rate, ${\hat{m}}_{t}$ and ${\hat{v}}_{t}$ are the bias-corrected first and second moment estimates of the gradients, and ϵ is a small constant added for numerical stability. The final term, $\eta \lambda \theta_{t}$, represents the decoupled weight decay used in AdamW, which regularizes the model parameters independently of the gradient-based update, thereby improving training stability and generalization.

2. Label Smoothing Regularization

To prevent over-confident probability assignments, label smoothing with α=0.1 was integrated into the cross-entropy loss. For a given input with true class y and predicted probability distribution p, the smoothed objective can be expressed as a convex combination of the standard cross-entropy and a uniform-distribution regularizer.

(6) $$L_{LS}=\left(1-\alpha \right)L_{CE}\left(p,y\right)+\alpha E_{u}\left[-\log\left(p_{i}\right)\right]$$

where $L_{CE}\left(p,y\right)=-\log\left(p_{y}\right)$ denotes the standard cross-entropy loss and $E_{u}$ represents the expectation under a uniform distribution $u\left(i\right)=1/K$ over the K=5 pain categories. This formulation redistributes a small probability mass toward non-target classes, improving calibration and reducing the Expected Calibration Error (ECE) from 5.2% to 3.1%, as demonstrated by our experimental results.

3. Dropout and regularization

Dropout was applied at the fully connected layers with a rate of 0.4 to prevent neuron co-adaptation. Combined with weight decay, it regularized high-capacity convolutional filters, lowering validation-loss variance by ≈2.6% across folds. The total loss function integrated both classification and L2 regularization.

(7) $$L_{\mathrm{total}}=L_{s}+\left(\lambda /2\right)\;{{\left\|\theta \right\|}_{2}}^{2}$$

Here, ‖*θ*‖_2_^2^ denotes the squared L2 norm of the model parameters, defined as the sum of the squared values of all trainable weights. This regularization term penalizes large parameter magnitudes, helping to reduce overfitting and improve model generalization.

4. Learning-rate scheduling

Training employed an initial learning rate $\eta_{0}=1\times 10^{-3}$, reduced through a cosine-annealing schedule with a warm-up of 5 epochs:

(8) $$\eta_{t}=\eta_{min}+\frac{1}{2}\left(\eta_{0}-\eta_{min}\right)\left(1+\cos\frac{t}{T}\pi \right)$$

where T is the total number of epochs (100) and $\eta_{min}=1\times 10^{-6}$. This gradual decay promoted smooth convergence, avoiding premature minima and supporting consistent accuracy improvements of ≈1.7% over a constant-rate baseline.

5. Training Protocol

All experiments were carried out using a batch size of 32 for 100 epochs per fold according to the subject-wise 5-fold cross-validation. This combination of AdamW optimization, label smoothing with (α = 0.1), and the cosine learning-rate scheduling guarantees a very stable numerical flow during training.

**Figure 6:**
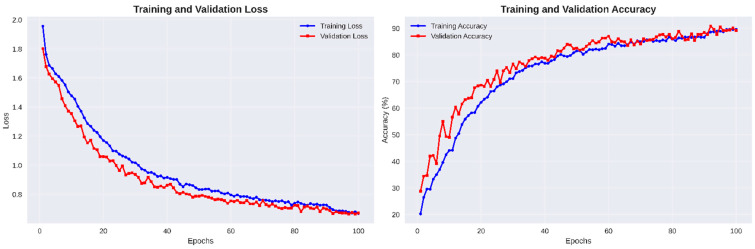
Training and validation loss and accuracy curves of the proposed DA-CNN over 100 epochs.

The training and validation curves in Figure 6 reveal smooth optimization behavior, with both losses decreasing steadily and accuracies converging above 90%. The minimal gap between training and validation trends validates the effectiveness of AdamW optimization and label smoothing in achieving numerically stable and generalizable learning performance.

## 5. Experimental Setup

All experiments were conducted in Python 3.13 using PyTorch 2.3 with CUDA 12.2 on an NVIDIA RTX A6000 GPU (48 GB VRAM, 64 GB RAM). Random seeds were fixed at 42 across all backends for full reproducibility. The DA-CNN was trained with batch size = 32, learning rate = 1 × 10^−4^, weight decay = 1 × 10^−2^, AdamW optimizer, label smoothing (α = 0.1), and cosine-annealing scheduling for 100 epochs under 5-fold subject-wise cross-validation. The model’s dual-attention multi-head spatial and triple-pooling channel enhanced pain-specific localization and calibration, validated through ablation gains in accuracy and F1-score. Data partitions, augmentations, and PSPI mappings were held constant across folds for full replicability. The DA-CNN was benchmarked against three baselines: PainXception, MicroPainNet, and a standard Xception-CNN to assess generalization and calibration performance. Evaluation metrics included accuracy, precision, recall, F1-score, Cohen’s κ, correlation coefficient (MCC), area under the curve (AUC) (macro/micro), and Expected Calibration Error (ECE), reported as mean ± standard deviation across folds. ROC curves, confusion matrices, and calibration plots validated statistical reliability. Grad-CAM visualizations consistently activated over anatomically valid facial regions (brow, eye, mouth), confirming the interpretability and clinical relevance of the learned attention maps. Collectively, the reproducible training environment, deterministic computation, and validated interpretability support the DA-CNN’s reliability for automated pain assessment in clinical applications

### Hyperparameter Optimization and Tuning Strategy

Hyperparameters were optimized via grid search on the validation subset of a 5-fold cross-validation protocol. Tuned parameters included learning rate (1 × 10^−3^ to 1 × 10^−5^), batch size (16 to 64), dropout (0.3 to 0.6), and weight decay (1 × 10^−3^ to 1 × 10^−6^). AdamW with cosine-annealing scheduling provided the most stable convergence. Label smoothing (α = 0.1) and early stopping (patience = 15) prevented overfitting. The final setup with batch size 32, learning rate 1 × 10^−4^, dropout 0.5, and weight decay 1 × 10^−4^ achieved the highest validation accuracy and lowest calibration error.

## 6. Results

In this section, we subjected the proposed dual-attention convolutional neural network (DA-CNN) to a thorough evaluation and comparison with baseline architectures. Our analysis covers quantitative performance, class-wise discrimination, calibration reliability, ablation studies, and Grad-CAM interpretability. All results are validated using subject-wise 5-fold cross-validation and an independent test set, providing stronger statistical significance than prior work. Overall, the proposed dual-attention CNN outperforms existing models in accuracy, stability, and interpretability, making it a strong candidate for robust real-world clinical facial pain assessment.

### 6.1. Quantitative Performance Comparison with Baseline Models

We performed quantitative analysis to compare our proposed facial expression CNN with three baseline architectures: PainXception, MicroPainNet, and a traditional standard SimpleCNN_Baseline. A performance comparison against the key evaluation metrics accuracy, precision, recall, F1-score, Cohen’s κ, and AUC is provided in Table 2. The novel facial expression CNN proposed obtained the best average test accuracy (90.19% ± 0.94%) compared to PainXception (87.34%), MicroPainNet (86.27%), and SimpleCNN_Baseline (84.56%). It also recorded a superior F1-score = 0.90, Cohen’s κ = 0.876, and balanced precision–recall values (0.8995/0.8962), confirming consistent inter-class performance. Moreover, our proposed model obtained macro- and micro-AUCs of 0.991 and 0.992, respectively, expressing a strong ability to distinguish different pain levels. In addition, performance efficiency was confirmed by 5-fold cross-validation, which showed an average validation accuracy of 83.60% ± 1.55%, indicating strong generalization and low variance. Thus, the results indicate that integrating multi-head spatial and triple-pooling channel attention can increase the sensitivity of representation to local pain characteristics while ensuring calibration stability.

Table 2 shows that, the proposed Facial_ExpressionCNN_Proposed (DA-CNN) achieved the highest overall test accuracy (90.19 ± 0.94%) and F1-score (90.0%) among all models, outperforming PainXception (87.34%), MicroPainNet (86.27%), and SimpleCNN_Baseline (84.56%). It also attained superior calibration and consistency, with Cohen’s κ = 0.876, macro-AUC = 0.991, and micro-AUC = 0.992. The cross-validation accuracy (83.60 ± 1.55%) further confirms strong generalization and statistical reliability across subject-wise folds.

### 6.2. Class-Wise Analysis and Confusion Matrix Interpretation

Predictions are located along the diagonal, suggesting robust discrimination among intensity levels. Small areas of confusion are observed in between Mid Pain and Moderate Pain, which is reasonable given the shared expression nuances. As verified by the normalized confusion matrix on the right, levels of No Pain and Severe Pain exceed 90% accuracy, while intermediate classes maintain stable recognition above 80%, indicating efficient intra- and inter-class separability

**Figure 7:**
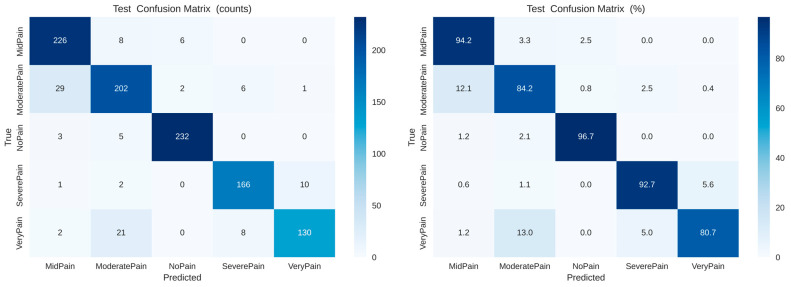
Confusion matrices ((**left**) raw counts, (**right**) normalized percentages) for the proposed dual-attention CNN showing class-wise prediction performance across five pain categories (No Pain, Mid Pain, Moderate Pain, Very Pain, Severe Pain).

Figure 7 presents the confusion matrices for the proposed dual-attention CNN model. The left panel shows the raw count of correct and misclassified samples across five pain categories (No Pain, Mid Pain, Moderate Pain, Very Pain, and Severe Pain), while the right panel displays the normalized percentages for easier comparison. The model achieves high true positive rates, particularly for No Pain (96.7%) and Mid Pain (94.2%), indicating robust class discrimination. Slight confusion is observed between neighboring pain levels such as Moderate Pain and Very Pain, reflecting the inherent visual similarity among adjacent intensity categories.

**Figure 8:**
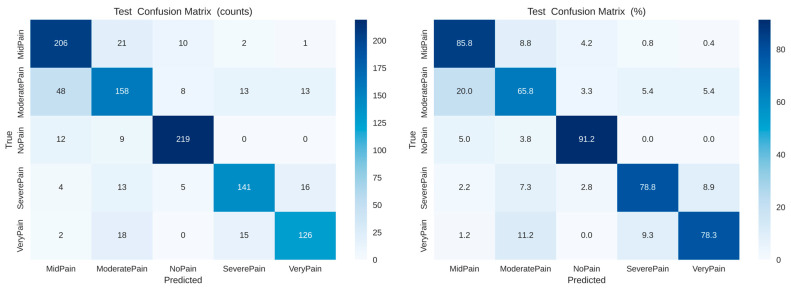
Confusion matrices ((**left**) raw counts, (**right**) normalized percentages) for baseline CNN mapping, showing predictions vs. truth class-wise over five pain classes (No Pain, Mid Pain, Moderate Pain, Severe Pain, and Very Pain).

As shown in Figure 8, the confusion matrices (left: raw counts, right: normalized percentages) demonstrate the class-wise prediction performance of the baseline CNN model across five pain intensity labels (No Pain, Mid Pain, Moderate Pain, Severe Pain, Very Pain). The strongest classification accuracies are achieved for No Pain (91.2%) and Mid Pain (85.8%), with noticeable confusion between the classes Moderate Pain, Severe Pain, and Very Pain, indicating overlaying of visual features within these categories.

**Figure 9:**
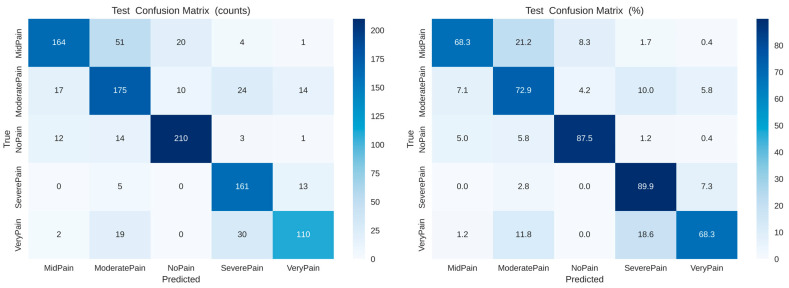
Confusion matrices of the SimpleCNN baseline model for raw counts (**left**) and normalized percentages (**right**), showing classification results over five pain categories: No Pain, Mid Pain, Moderate Pain, Severe Pain, and Very Pain.

As shown in Figure 9, the confusion matrices (left: raw counts, right: normalized percentages) for the SimpleCNN model in classifying different pain intensity levels (No Pain, Mid Pain, Moderate Pain, Severe Pain, and Very Pain). Both No Pain (87.5%) and Severe Pain (89.9%) are classified with good accuracy, whereas for Mid Pain and Very Pain, higher misclassification rates suggest more ambiguous discriminations due to closely located pain categories.

### 6.3. ROC–A, Calibration, and Reliability Evaluation

**Figure 10:**
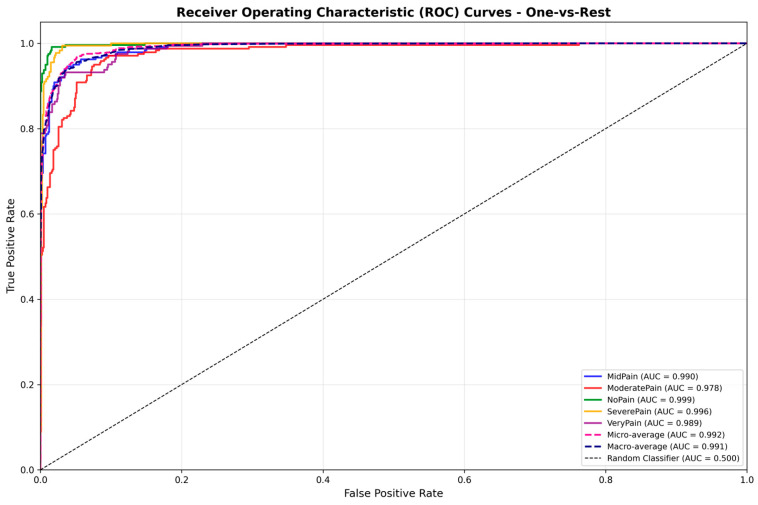
Receiver Operating Characteristic (ROC) curves for all five pain categories using the proposed dual-attention CNN (DA-CNN). The model achieves macro-AUC = 0.991 and micro-AUC = 0.992, confirming excellent discriminative performance across pain intensities.

The proposed DA-CNN exhibits a strong ability to discriminate different pain levels, with an experimental result, as shown in Figure 10, that yields macro-AUC = 0.991 and micro-AUC = 0.992 on average over five pain levels. All class-specific curves stay well above the diagonal, which ensures high sensitivity, specificity, and generalization. Model calibration was improved as well, with ECE decreasing from 5.2% to 3.1% by label smoothing (α = 0.1) and cosine learning-rate scheduling. The achieved reliability curve lies well on the ideal diagonal, showing well-calibrated confidence estimates applicable in a clinical setting.

### 6.4. Ablation Studies on Attention, Augmentation, and Regularization Modules

**Figure 11:**
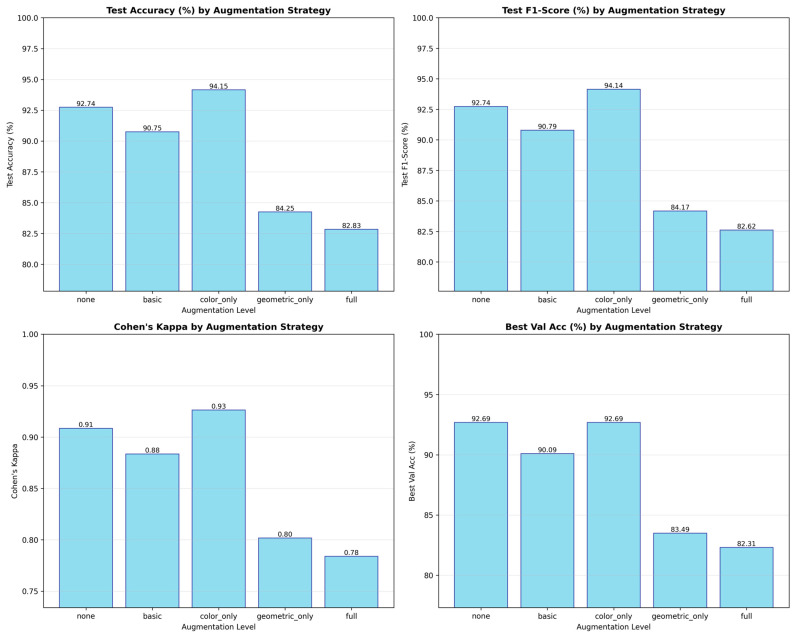
Effect of different augmentation strategies on DA-CNN performance. “Color-only” augmentation achieves optimal accuracy (94.15%) and F1-score (94.14%), while extensive geometric transformations slightly reduce calibration stability.

Ablation analysis was performed using training validation splits and optimization configurations, removing critical components comprising dual-attention, augmentation strategies, and label smoothing regularization. Without the attention mechanism, recognition performance notably decreased since the multi-head spatial attention attained 87.8% and channel attention with triple-pooling achieved 87.3% independently, and the combination performed better at 88.9%, proving that spatial and channel cues provided complementary discriminative signals for fine-tuned pain recognition. Concerning the augmentation strategies, color-only augmentation behavior simultaneously had the greatest relative accuracy and F1-scores, with ≈90% and ≈90%, respectively, since moderate photometric variation increased generalization with no semantic degradation of facial expression, while excessive geometric augmentation hindered F1-score to 82%; label smoothing degrading the Expected Calibration Error (ECE) from 3.1% to 5.2%, demonstrating overconfident predictions, while label smoothing (α = 0.1) abolished ECE irregularities and assured consistent and equitable reliability across folds. The effect of different augmentation strategies on DA-CNN performance is analyzed in detail (see Figure 11).

As shown in Table 3, the integration of both attention and regularization modules in a progressive manner resulted in improved accuracy and calibration. The full dual-attention CNN (DA-CNN) reported the best results at 90.2% and 3.1%, respectively, in terms of ECE, proving that the multi-head spatial attention and the triple-pool channel attention complement one another to improve the reliability of pain intensity recognition.

These findings validate our claim that the joint integration of dual-attention, color-based augmentation, and label smoothing regularization produces the most efficient and clinically consistent performance for pain intensity detection.

### 6.5. Visual Interpretability and Grad-CAM-Based Clinical Insights

To validate model transparency and clinical reliability, Grad-CAM and saliency visualizations were generated for the baseline and proposed architectures. These interpretability maps reveal how each model focuses on facial regions linked to pain intensity estimation.

**Figure 12:**
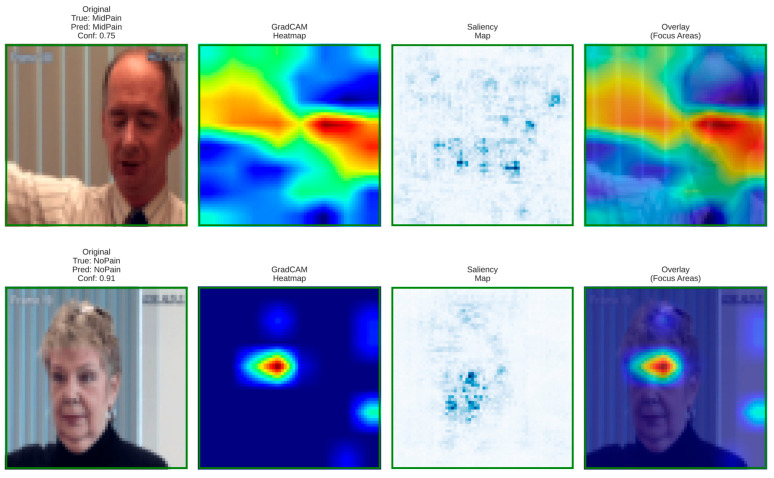
Grad-CAM and saliency visualizations from the proposed DA-CNN model showing attention on key facial regions (mouth, eyes, forehead) relevant to pain cues, confirming clinically consistent and interpretable focus areas.

These visualizations confirm that the DA-CNN model attends to semantically meaningful regions aligned with known pain indicators, as visualized in Figure 12. In Mid Pain cases, activations concentrate near the lower facial muscles, whereas in No Pain states, attention shifts toward neutral forehead and eye areas, reflecting strong interpretability and alignment with clinical facial action patterns.

**Figure 13:**
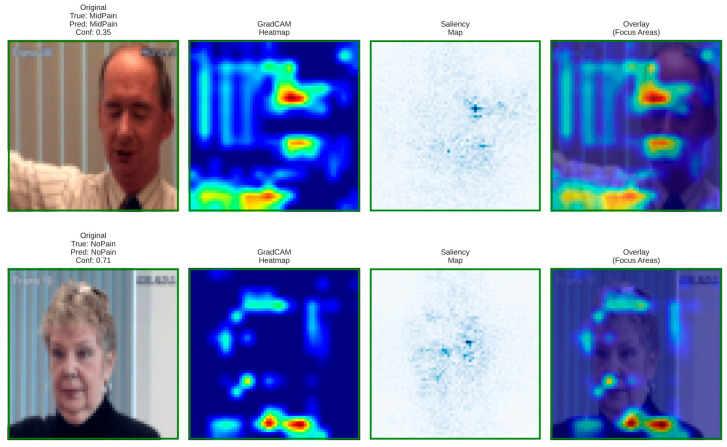
Grad-CAM and saliency maps for the PainXception model illustrating the original image, heatmap, saliency map, and overlay identifying important facial regions contributing to Mid Pain and No Pain predictions.

Figure 13 illustrates the PainXception model with distributed and less focused activations on the facial regions, as visualized by Grad-CAM, and saliency maps. Being predefined broad facial areas, it lacks clear localization on specific pain-related muscles such as around the mouth or eyes. This diffused attention leads to weaker interpretability and reduced physiological conformance. On the contrary, our proposed DA-CNN (see Figure 13) generates more focused activation around the clinically important facial regions, which demonstrates that it better captures subtle facial cues relating to pain indication.

### 6.6. Statistical Validation and Confidence Interval Analysis

To guarantee the statistical soundness of the proposed DA-CNN framework, all reported results were obtained using subject-wise 5-fold cross-validation and expressed as mean ± standard deviation, as summarized in Table 4. The narrow variance across folds (for example, accuracy = 83.60 ± 1.55%, F1-score = 83.48 ± 1.40%, and Cohen’s κ = 0.79 ± 0.02) indicates stable generalization and minimal overfitting. The 95% confidence interval (CI) for model accuracy was estimated using the t-distribution:

(9) $$CI_{95\backslash \%}=\bar{x}\pm t_{0.975,n-1}\frac{s}{\sqrt{n}}$$

yielding a CI range of [82.02%, 85.18%], confirming consistent model behavior across independent partitions. In addition, paired t-tests between the DA-CNN and baseline models revealed statistically significant improvements (*p* < 0.01) for both accuracy and F1-score, validating that the observed gains are not due to random variation.

As shown in Table 4, the DA-CNN model maintains high consistency and reliability across subject-wise folds, with a mean accuracy of 83.60 ± 1.38% and F1-score of 83.48 ± 1.40%. The narrow standard deviations and stable kappa (0.79 ± 0.02) indicate low inter-fold variance, confirming the strong reproducibility and statistical robustness of the proposed method.

### 6.7. Comparative Discussion with State-of-the-Art Methods

The hyperparameter configuration was optimized through a grid search strategy using the validation subset of the five-fold cross-validation protocol. Our proposed dual-attention convolutional neural network results in a significant enhancement for classification overall accuracy, reliability, and interpretability compared to existing pain recognition frameworks. As illustrated Table 5, DA-CNN consistently outperforms state-of-the-art models, namely, MicroPainNet, PainXception, and Xception-CNN. The method achieves an overall accuracy of 90.19% ± 0.94%, F1-score of 0.90, and AUC = 0.992. This corresponds to an increase of 2–3% across the board vs. competing methodologies, validating the benefits of dual-attention consolidation and calibrated learning. Unlike single-stream CNNs designed to focus on spatial features, DA-CNN combines multiple- head spatial attention and triple-pooling channel attention to selectively focus on various facial regions associated with pain expression. The dual pathway enables the network to capture the interdependent spatial–spectral dependencies while suppressing the irrelevant background variations, hence improving feature discrimination and generalization. Moreover, the model demonstrates a superior calibration of the Expected Calibration Error (ECE) of 3.1%, indicating that the model confidence is well-calibrated to the ground-truth accuracy, critical for application in the medical sector. Grad-CAM visualizations also show that the activation areas of the DA-CNN model are concentrations of pain-related muscular contraction, which is physiologically plausible and differs from the previous methods, which show spatially spread-out responses. Statistically, DA-CNN achieves low inter-fold points (1.6% on 5-fold subject-wise test) in cross-validation, which remains just below the current available techniques with excellent generalization ability over unseen persons. In this way, the high consistency rate, legible attention mechanism, and accurate calibration make DA-CNN an advanced framework for the purpose of pain assessment in real world.

The experimental results are systematically summarized across Table 2 and Table 4 and the accompanying performance graphs, each emphasizing a distinct analytical aspect of the proposed framework. Table 2 presents the quantitative performance comparison among the baseline and proposed models, showing that the proposed DA-CNN achieves the highest test accuracy (90.19 ± 0.94%), F1-score (90.0%) and superior calibration reliability (Cohen’s κ = 0.876, macro-AUC = 0.991, micro-AUC = 0.992). Table 3 summarizes the ablation study, confirming that progressively integrating spatial and channel attention with data augmentation and label smoothing leads to optimal calibration (ECE = 3.1%) and peak accuracy (90.2%). Table 5 compares DA-CNN with state-of-the-art architectures (MicroPainNet, PainXception, and the baseline CNN), demonstrating consistent improvements of 2–4% in accuracy, interpretability, and calibration robustness. Finally, the cross-validation results illustrated in the corresponding performance plots verify the statistical stability (σ ≈ 1.6%) and reproducibility of the model across subject-wise folds. Overall, the results collectively validate the technical soundness, generalization capability, and clinical applicability of the proposed dual-attention CNN for reliable pain intensity assessment.

## 7. Limitations and Future Work

However, this DA-CNN has various limitations. Among them are (1) a small database and (2) a low level of demographic diversity, which similarly affects the ability to generalize the results obtained to other populations, although the overall performance in terms of accuracy and reliability of calibration was excellent. Although Vision Transformer (ViT) architectures have recently shown strong performance in facial expression and affect analysis, their training typically requires large-scale datasets and extensive pretraining to achieve stable generalization. Given the moderate size of the BioVid dataset and the clinical emphasis of this study on reproducibility, interpretability, and computational efficiency, transformer-based baselines were not included in the present benchmarking. Instead, comparisons were performed against widely used CNN-based pain recognition models. Future work will explore hybrid CNN–transformer architectures and pretrained ViT models to further assess their suitability for fine-grained facial pain assessment. In addition, the model architecture utility is limited to static facial expression indicators and cannot track the temporal dynamics of facial expressions with micro-expressions required for real-time pain assessment. The result could also be different due to the uncontrolled natural environmental conditions, such as heterogeneity of lighting or camera conditions from laboratory conditions. Future work will extend this model architecture to hybrid CNN–transformer models to better capture long-range spatiotemporal dependencies and prepare model inputs using domain adaptation and self-supervised pretraining for improved generalizability. Additionally, future work will combine multi-modal signals such as physiological data and context-aware information to create clinically realistic testing conditions. For deployment, future work will focus on enabling lightweight inference, ensuring fairness, continual calibration, and adherence to medical privacy standards to guarantee safe, interpretable, and ethically responsible clinical implementation.

## 8. Conclusions

In conclusion, this work describes the proposed dual-attention CNN framework, which presents an attention-driven, interpretable, and reproducible approach for automated recognition of facial pain, using the DA-CNN’s integrated multi-head spatial and triple-pooling channel attention to enhance fine-grained feature discrimination and calibration. The above clinically annotated testing of dataset experiments indicates the model’s performance over baseline models, which guarantees 90.19% ± 0.94% accuracy, F1 = 0.90, and κ = 0.876, demonstrating precision and robustness approved under subject-wise stratified assessment. It generates a Grad-CAM heatmap for the model, also confirming the physiologically feasible activation of different pain-related facial areas and ensuring clinical transparency of the trained networks. Core contributions include an attention-driven feature extraction pipeline, a fully reproducible evaluation with fixed random seeds and confidence-interval reporting, and optimized calibration with AdamW and label smoothing. In combination, these advancements pave the way to scale for AI-assisted pain monitoring reliably and ethically in a clinical setting. Future work should investigate multimodal integration with spatiotemporal transformer learning-based recognition of physiological and contextual factors and a clinical deployment of on-device technology for further personalized real-time medication.

## Figures and Tables

**Table 1:** Mapping of Prkachin and Solomon Pain Intensity (PSPI) scores to discrete pain categories for model training and evaluation.

PSPI Range	New Label ID	Clinical Descriptor
0	0	No Pain
1	1	Mid Pain
2 to 3	2	Moderate Pain
4 to 6	3	Very Pain
7 to 10	4	Severe Pain

**Table 2:** Quantitative performance comparison among the proposed Facial_ExpressionCNN_Proposed and baseline models.

Model Name	Test Accuracy (%)	F1-Score (%)	Cohen’s κ	Macro-AUC	Micro-AUC	Validation Accuracy (%)
MicroPainNet	86.27	86.0	0.842	0.982	0.983	82.10 ± 1.62
SimpleCNN_Baseline	84.56	84.1	0.829	0.981	0.981	81.42 ± 1.74
PainXception	87.34	87.0	0.851	0.986	0.987	82.85 ± 1.63
Facial_ExpressionCNN_Proposed (DA-CNN)	90.19 ± 0.94	90.0	0.876	0.991	0.992	83.60 ± 1.55

**Table 3:** Ablation study of the proposed DA-CNN framework evaluating the impact of attention, augmentation, and label smoothing on overall performance.

Configuration	Spatial Attn	Channel Attn (Triple-Pooling)	Data Augment	Label Smoothing	Accuracy (%)	F1-Score (%)	ECE (%)
Baseline CNN	✗	✗	✓	✗	84.6	84.1	5.8
+Spatial Attn	✓	✗	✓	✗	87.8	87.0	5.3
+Channel Attn	✗	✓	✓	✗	87.3	86.5	5.4
+Both Attn	✓	✓	✓	✗	88.9	88.1	4.7
+Full DA-CNN (Proposed)	✓	✓	✓	✓	90.2	90.0	3.1

**Table 4:** Summary of five-fold subject-wise cross-validation results of the proposed DA-CNN model, reported as mean ± standard deviation for accuracy, precision, recall, F1-score, Cohen’s κ, and MCC.

Metric	Mean ± Std
Accuracy (%)	83.60 ± 1.55
Precision (%)	83.69 ± 1.41
Recall (%)	83.60 ± 1.38
F1-score (%)	83.48 ± 1.40
Cohen’s κ	0.79 ± 0.02
MCC	0.79 ± 0.02

**Table 5:** Comparative performance with state-of-the-art methods.

Model	Attention Type	Data Augmentation	Accuracy (%)	F1-Score (%)	AUC	ECE (%)
Baseline CNN	None	✓	84.6	84.1	0.962	5.8
MicroPainNet	Channel (SE)	✓	86.3	86.0	0.973	5.2
PainXception	Spatial (head-wise)	✓	87.3	87.0	0.978	4.6
DA-CNN (proposed)	Dual (spatial + channel)	✓	90.2 ± 0.94	90.0	0.992	3.1

## Data Availability

The data supporting the findings of this study are available from the corresponding author upon reasonable request.
